# Genetics of male infertility: status and prospects

**DOI:** 10.1186/1471-2164-15-S2-O25

**Published:** 2014-04-02

**Authors:** Eberhard Nieschlag, Frank Tüttelmann, Mohammed A Beg

**Affiliations:** 1 Center of Reproductive Medicine and Andrology, University of Münster, 48129 Münster, Germany; 2 Institute of Human Genetics, University of Münster, 48129 Münster Germany; 3 Center of Excellence in Genomic Medicine Research, King Abdulaziz University, Jeddah, Kingdom of Saudi Arabia

## 

The incidence of infertility is about 15% in couples of childbearing age. In half of these couples a fertility problem exists on the male side. However, causes of male infertility are still to a large extent unknown. Today, in about 20% of patients with azoospermia or severe oligozoospermia genetic causes have been identified and further progress is expected from genetic investigations.

The Klinefelter syndrome represents the most frequent form of human aneuploidies and male infertility. The high incidence of 1:500 in the general male population increases to 1:50 in infertility centres. Ninety percent of the affected men show azoospermia. In recent years it became possible to obtain viable sperm by testicular biopsy, followed by TESE/ICSI, resulting in pregnancies. Since these sperm may derive from isolated spermatogonia with a regular karyotype, most of the children have normal karyotypes.

AZF a, b, and c deletions of the Y chromosome are found in men with severe infertility. The incidence among patients with very low sperm counts or azoospermia varies between 2.5 and 10 %. From patients with AZFc deletions sperm may be retrieved from testicular biopsies and induction of pregnancy with these sperm is possible by applying TESE/ICSI. Patients with AZF a and b deletions mostly have complete Sertoli-cell-only-syndrome (SCOS) and have no chances for paternity. Male children of men with microdeletions in the AZF region have the same deletions as their fathers and are likely to be infertile themselves.

Mutations of the CFTR gene affect functioning of the chloride ion channels in the epithelial cell membranes leading to cystic fibrosis and congenital bilateral absence of the vas deferens (CBAVD). CBAVD may occur as a minimal form of cystic fibrosis (CF). Before TESE/ICSI is applied, the status of the female partner needs to be determined in order to avoid offspring suffering from CF.

A number of genes mutated in isolated hypogonadotropic hypogonadism (IHH) and Kallmann syndrome have been identified Oligozoospermic men with combined single nucleotide polymorphism of *FSHR* 2039A>G and *FSHB*-211G>T may benefit from FSH therapy.

Although men may remain fertile lifelong, a slow decline of reproductive capacities occurs beyond the age of 45 and specific gene mutations e.g. of the FGFR2 and FGRF3 genes causing Apert syndrome and achondroplasia may increase, affecting offspring.

Investigations into epigenetics such as methylation abnormalities, transcriptome analysis as well as single and combined single-nucleotide polymorphism genotypes and whole genome analysis in individual sperm may lead to the identification of further genetic associations with infertility.

